# Development-Associated Genes of the Epidermal Differentiation Complex (EDC)

**DOI:** 10.3390/jdb12010004

**Published:** 2024-01-15

**Authors:** Karin Brigit Holthaus, Leopold Eckhart

**Affiliations:** Department of Dermatology, Medical University of Vienna, 1090 Vienna, Austria

**Keywords:** epidermis, keratinocytes, cornification, periderm, subperiderm, loricrin, skin barrier, stratum corneum, chicken, evolution

## Abstract

The epidermal differentiation complex (EDC) is a cluster of genes that encode protein components of the outermost layers of the epidermis in mammals, reptiles and birds. The development of the stratified epidermis from a single-layered ectoderm involves an embryo-specific superficial cell layer, the periderm. An additional layer, the subperiderm, develops in crocodilians and over scutate scales of birds. Here, we review the expression of EDC genes during embryonic development. Several EDC genes are expressed predominantly or exclusively in embryo-specific cell layers, whereas others are confined to the epidermal layers that are maintained in postnatal skin. The S100 fused-type proteins scaffoldin and trichohyalin are expressed in the avian and mammalian periderm, respectively. Scaffoldin forms the so-called periderm granules, which are histological markers of the periderm in birds. Epidermal differentiation cysteine-rich protein (EDCRP) and epidermal differentiation protein containing DPCC motifs (EDDM) are expressed in the avian subperiderm where they are supposed to undergo cross-linking via disulfide bonds. Furthermore, a histidine-rich epidermal differentiation protein and feather-type corneous beta-proteins, also known as beta-keratins, are expressed in the subperiderm. The accumulating evidence for roles of EDC genes in the development of the epidermis has implications on the evolutionary diversification of the skin in amniotes.

## 1. Introduction: The Protective Functions of the Epidermis

The skin of amniotes prevents excessive loss of water in a dry environment and protects against mechanical, physical and biological threats. These functions are mediated by the epithelium of the skin, the epidermis, and in particular by the outermost layer of the epidermis, the cornified layer or stratum corneum. The epidermis is built by keratinocytes which undergo terminal differentiation, leading to their conversion into corneocytes, which are the cellular components of the stratum corneum. While the epidermis is continually renewed by cell proliferation, differentiation and cell death in adult organisms [1,2], the embryonic development of the epidermis involves special processes, such as the formation of a periderm, that are not active in the adult epidermis [3,4,5].

The molecular regulation of the epidermis is of great interest for evolutionary biology and medicine. Numerous specializations of skin development and regeneration have underlain the adaptation to diverse environments from dry deserts to the sea and the evolution of skin appendages, such as scales, hair, feathers and mammary glands, which are the characteristic features of major taxa of terrestrial vertebrates [6,7]. Dysregulations of epidermal homeostasis and stress responses cause highly prevalent skin diseases such as atopic dermatitis, which affects more than 10% of newborns [8]. Here, we review an important aspect of skin biology that has been uncovered by recent publications, namely the difference between the genetic regulation of the epidermis during development and during postnatal life.

## 2. Epidermal Differentiation in Mammals

The basic principles of the epidermal differentiation process have been identified in mammalian skin [9,10,11,12]. Keratinocytes proliferate in the basal layer, where also the stem cells reside, and subsequently move upwards into the suprabasal epidermal layers. Upon delamination, keratinocytes stop proliferation and undergo terminal differentiation, which depends on specific changes of gene expression, such as the upregulation of keratins K1 and K10, involucrin (IVL), small proline-rich proteins (SPRRs), transglutaminases and proteases [9,10]. Several differentiation-associated genes, such as loricrin and filaggrin, are expressed specifically in the granular layer of mammalian epidermis [13]. The final step of differentiation is the conversion of living keratinocytes into dead corneocytes that build the cornified layer (stratum corneum) on the skin surface [11,14,15]. This mode of cell death is called cornification or corneoptosis [16] and involves the breakdown of the nucleus and other cell organelles, while the cross-linking of proteins leads to the formation of the so-called cornified envelope underneath the cell membrane. The crosslinking of proteins requires the elevation of the intracytoplasmic concentration of calcium ions [17], which enables calcium-dependent transglutaminases TGM1, TGM3 and TGM5 to introduce N^ɛ^-(-glutamyl)lysine isopeptide bonds between proteins [11]. Ultimately, the cell membrane is replaced by inter-cellular lipids that fill the space between corneocytes and limit the diffusion of water through the stratum corneum. Cornified envelopes consist of cross-linked proteins, such as involucrin, loricrin, envoplakin, periplakin, SPRRs, filaggrin and keratins [11,13,15,18]. Desmosomes connect keratinocytes during differentiation and become modified by the interaction with corneodesmosin to form corneodesmosomes, which are the connections between corneocytes. Corneodesmosomes provide mechanically stable links between corneocytes and thereby establish the resilience of the stratum corneum. The proteolytic degradation of corneodesmosomes in the outermost layers of the stratum corneum, which is controlled by kallikreins and kallikrein inhibitors, leads to the desquamation of corneocytes [19].

The keratin cytoskeleton of keratinocytes undergoes major changes before and during keratinization. Filaggrin is considered a major regulator of this process because keratin filaments aggregate in its presence in vitro [20], and mutations of the filaggrin genes are associated with defects of the skin barrier. The precursor protein profilaggrin is synthesized in the granular layer where it is phosphorylated and located, at least when analyzed by histological procedures, in keratohyalin granules, which are the distinguishing characteristic of granular cells. Dephosphorylation, deimination and limited proteolysis control the maturation of filaggrin [21,22,23].

## 3. Epidermal Differentiation in Sauropsids

Sauropsids are the phenotypically diverse sister group of mammals, together forming the clade of amniotes. Extant sauropsids comprise Lepidosauria (Rhynchocephalia, represented by the tuatara, and Squamata, including geckos, lizards and snakes) and Archelosauria (turtles, crocodilians and birds). Numerous specialized cornified structures are present on the skin of sauropsids: for example, cornified scales of various shape in all reptiles, adhesive setae on the feet of geckos, scutes of the turtle shell, rhamphotheca of turtles and feathers of birds. Epidermal differentiation in squamates involves the regeneration and shedding of the outer layers of the epidermis, which is associated with a complex histological structure of the epidermis [24]. All these features of the sauropsidian epidermis are expected to depend on the molecular components of cornified structures and molecular mechanisms of regulation that are unique to sauropsids or subclades of sauropsids. Many of the proteins that build cornified epidermal cells of sauropsids have been identified by recent research, whereas the regulatory processes are largely unknown at present.

As the current knowledge on the skin of sauropsids has been reviewed extensively [7,25,26], we highlight only a few aspects here. Basic principles of epidermal differentiation, such as cell proliferation in the basal layer, changes in gene expression during movement through suprabasal layers and cell death with massive protein cross-linking (cornification), are equivalent to those in mammalian epidermis. Soft cornification, leading to a pliable skin surface like in mammals, occurs in the epidermis between feather follicles of birds, on regions of the neck and limbs of turtles, and in the hinge and inner segments of scales in all sauropsids. By contrast, hard cornification, leading to rigid scales, scutes, carapace, feathers etc., is very prominent in reptiles and birds. Of note, the morphogenesis of hard cornified skin appendages such as feathers, but also hair, requires complex regulation that goes beyond the control of epidermal differentiation discussed in the present review. Importantly, the existence of sauropsid-specific modes of epidermal differentiation mainly characterized at the histological level correlates with the existence of sauropsid-specific epidermal differentiation genes that have been identified and partially characterized [27,28,29,30,31,32,33,34,35,36].

## 4. Embryonic Development of the Epidermis in Amniotes

The epidermis is continuously renewed during postnatal life, but its initial stratification during embryonic development requires additional steps of epithelial differentiation [37]. In amniotes, the embryo is surrounded by the amniotic fluid, and the ectoderm gives rise to the embryonic epidermis. At first, the embryo is only covered by a monolayer of highly proliferative ectodermal cells. In the next step of skin development, the embryonic epidermis becomes bilayered with a transient protective layer called periderm covering the basal layer [38]. The limbs and tail are the first sites of periderm appearance, from which it extends to all other sites of the body surface in mice [39,40]. The periderm consists of flattened and tightly connected cells that are lost before birth [3,39,41,42]. While the periderm is often referred to as a single-layered epithelium, it is actually bilayered in rats [43] and mice [44] as well as reptiles [45]. In both single-layered and bi-layered periderm, the cells on the surface form microvilli.

After the formation of the periderm, epidermal stratification occurs through asymmetric cell division in the basal layer. The embryonic superbasal layers are not well characterized in early development but assume properties similar to the spinous layer and later by the granular layer as found in postnatal epidermis [5,46]. The developing epidermis is immature until the formation of tight junctions in the granular layer and the initiation of cornification. When the definitive cornified layer is formed, the periderm loses its function as a protective layer. Then, periderm cells undergo cornification, and the periderm detaches from the surface of the skin. These last steps of periderm cornification are coordinated with the maturation of the epidermis, as an impairment of epidermal cornification leads to the aberrant retention of the periderm in mutant mice [47].

The periderm of birds displays a special cytological feature: periderm granules [26]. These membrane-less granules of diameters between 100 nm and several micrometers are eosinophilic on histological sections. In samples prepared for ultrastructural studies, periderm granules appear to contain filaments 20–60 nm in diameter [48]. Recent studies on keratohyalin granules, which differ biochemically from periderm granules, but also appear in differentiated epithelial cells, have identified liquid–liquid phase separation as the main driver of granule formation. It is conceivable that a similar phenomenon occurs in the periderm and that the ribbon-like aggregations may be largely an artifact of tissue fixation. Peridermal granules in the transient embryonic layers disappear during the later stages of development once the cornified layer is formed [26,49,50].

**Figure 1:**
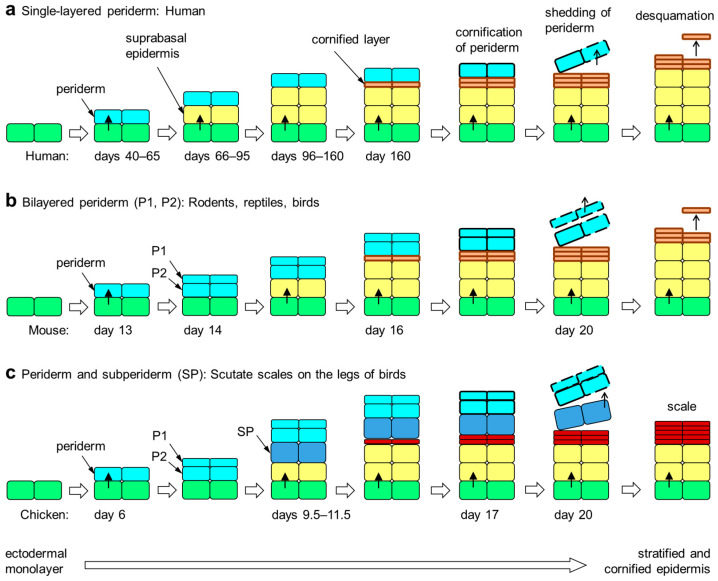
Embryonic development of the epidermis in amniotes. The periderm is a transient layer of epithelial cells covering the epidermis during its initial stratification in the embryo. Upon formation of the cornified layer of the epidermis, peridermal cells also undergo cornification. Subsequently, the periderm is shed from the surface. The periderm is either single-layered (**a**), two-layered (**b**) or multi-layered (not shown). Developing scutate scales of birds are covered by a subperiderm in addition to the bilayered periderm (**c**) [51,58]. The timing (days of embryonic development) of epithelial stratification events in selected species [5,44,51,59] is shown in each panel. Colors indicate the differentiation states of epithelial cells: green, basal layer keratinocytes; yellow suprabasal keratinocytes; orange, cornified keratinocytes of the stratum corneum; red, cornified keratinocytes of cornified scales; light blue, periderm; dark blue, subperiderm.

While in all sauropsids two periderm layers (outer/primary and inner/secondary) are present [45,51], in birds and crocodilians, which together form the clade Archosauria, a unique peridermal derivative, termed the subperiderm, has been reported [52,53,54,55,56]. To avoid confusion, we have to point to inconsistent use of this term in the literature with some papers referring to the inner layer of periderm as subperiderm [57] and others referring to the outer layers of the epidermis proper as subperidermal layers. Here, we follow the definition of the subperiderm by Roger H. Sawyer [51]. The subperiderm is the innermost layer of embryo-specific epithelial cells and is located between the inner periderm and the outermost layer of the immature epidermis. Although, to the best of our knowledge, experimental evidence is not available, the cells of the subperiderm are derived from precursors in the periderm. In contrast to the flattened cells of the periderm, subperidermal cells are thick and appear mechanically robust in histological sections. At the ultrastructural level, they are distinguished from the inner periderm by the absence of periderm granules, and at the molecular level, they contain specific corneous beta-proteins, which are also known as beta-keratins similar or identical to proteins of feathers [51]. A subperiderm develops on top of scutate scales on the legs of birds and on other types of scales in alligators and crocodiles [56] (Figure 1).

Specific body sites of sauropsids are covered by multiple transient layers of epithelial cells during development. For instance, the cornified egg tooth, also known as caruncle, is surrounded by a multilayered periderm in birds [50,60] and turtles [61]. The carapace of turtles is covered by several embryonic epithelial layers, which are shed at hatching [45,62].

## 5. The Epidermal Differentiation Complex (EDC)

**Figure 2:**
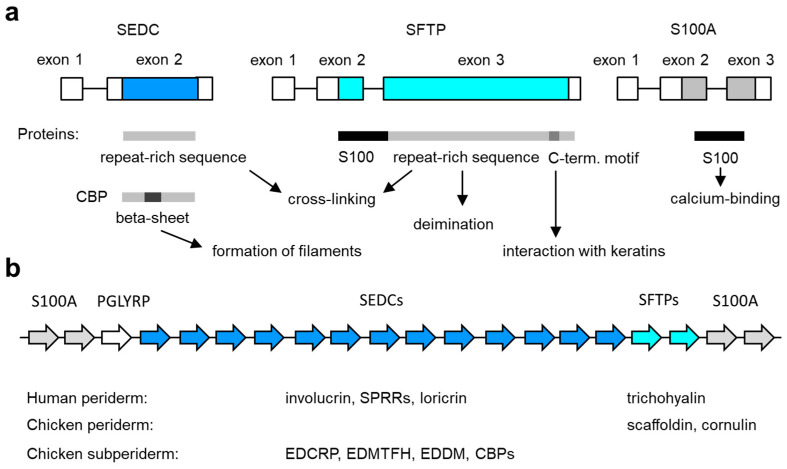
Genes of the epidermal differentiation complex (EDC). (**a**) Exon–intron structure of the main types of EDC genes. The basic structure of the encoded proteins and their functions are schematically depicted below. C-term., carboxy-terminal; SEDC, single-coding exon EDC gene; SFTP, S100 fused-type protein. (**b**) Arrangement of genes in the EDC. The relative arrangement of EDC gene types (indicated by arrows of different color, labeled at the top) in amniotes is depicted schematically. The number of genes in the diagram does not represent a particular species. The EDC contains 39 SEDC and 7 SFTP genes in humans [72] and 100 SEDC and 2 SFTP genes in the chicken [35]. The expression profile of SEDC and SFTP genes in the embryonic periderm and subperiderm is indicated below the schematic. See the main text for information on individual genes.

The EDC is a cluster of genes that are expressed during the terminal differentiation of keratinocytes [13,63]. First identified in humans on chromosome 1q21 [64,65] and later on chromosome 3q of the mouse, this locus is generally conserved in mammals [63,66] and sauropsids [31]. The EDC is delimited by S100A genes at both ends. One or two genes encoding peptidoglycan recognition proteins (PLGYRPs) are present close to the *S100A9* gene in the EDC of amniotes [31]. The sauropsid-specific gene *EDKM* (epidermal differentiation protein containing a KKLIQQ motif) is located next to *PLGYRP3* [31]. The remaining genes are classified into two types, single-coding exon EDC (SEDC) genes and S100 fused-type protein (SFTP) genes, which will be described in detail below (Figure 2).

The arrangement of genes in the EDC, as depicted schematically in Figure 2, is conserved in amniotes [31] and partially similar to a less complex gene cluster in amphibians [67]. The relative position of genes is important for the regulation of EDC gene expression, which depends on proximal promoters, distal enhancers and chromatin interaction networks [66,67,68,69,70,71]. The mechanisms of EDC gene regulation during keratinocyte differentiation and epidermal development and aging are proposed to be critical for the function of the EDC.

### 5.1. SEDCs

Single-coding exon EDC (SEDC, in some publications also referred to as simple EDC) genes have two exons of which the first contains only a non-coding sequence and the second codes for a protein. Exon 1 of most SEDC genes is preceded by a canonical TATA box [73], indicating that the expression of SEDCs is inducible and not required for constitutive cell functions [74]. The amino acid sequences of SEDC-encoded proteins are highly diverse, but there are several conserved features. Many SEDC proteins contain a conserved sequence motif close to their amino-terminus (consensus sequence QCKQPCxPP) and another imperfectly conserved motif close to their carboxy-terminus (consensus sequence QQxKQPXXWPxxxK-stop) [31,34]. Most SEDC proteins contain multiple tandem sequence repeats, and their amino acid composition is biased toward either glycine, proline or cysteine [31].

Although all SEDC genes are homologous, it is difficult to determine which SEDC genes of two species are orthologous, which means that they are more closely related to each other than to any other SEDC gene of the two species. There is good support for the orthology of loricrin in mammals and sauropsids [31], and possibly mammalian SPRRs and specific proline-rich proteins of sauropsids are also orthologs [36]. In contrast, a gene partially identified in the chicken and termed involucrin [30] is likely not closely related to mammalian involucrin [31]. Accordingly, new names had to be given to these sauropsid SEDC genes when a large number was identified by comparative analysis in the genomes of chicken and green anole lizard [31]. As detailed data on expression patterns and functions were not available at that time, the names refer to the location in the EDC, indicated by the letters ED for epidermal differentiation, and amino acid sequence features of the proteins encoded by these genes. Accordingly, EDPE stands for Epidermal Differentiation protein rich in Proline and glutamic acid (E) [31].

### 5.2. SFTPs

SFTPs have three exons, of which the second and third contain the protein-coding sequence [31]. Like in SEDC genes, the first exon of SFTPs is non-coding (Figure 2a) [31,63]. SFTPs have an amino-terminal S100 domain containing two Ca^2+^-binding EF-hand motifs. The S100 domain is followed by a segment of low-sequence complexity of 200–4000 residues, which is likely to result in an intrinsically disordered structure [75]. Both the length and amino acid sequence of this segment varies greatly between different SFTPs, but, in majority of SFTPs reported so far, this segment contains sequence repeats, and its amino acid composition is biased toward arginine and glutamic acid (e.g., in scaffoldin and trichohyalin, see below) or glycine and serine (e.g., filaggrin). Glutamine residues, potentially serving as sites of transglutamination, are frequent in all SFTPs [63]. A short sequence motif with the core sequence SPLY(E/D)Y is present close to the carboxy-terminal of most SFTPs. This motif has been proposed to mediate the binding of SFTPs to keratins [76,77,78].

## 6. Expression and Function of EDC Genes in Epidermal Development

### 6.1. EDC Genes Expressed in the Embryonic Precursor of the Postnatal Epidermis

While the periderm and subperiderm are specifically embryonic layers of the epidermis, the basal, spinous, granular and cornified layers of stratified epidermis are present in the late embryonic skin and in postnatal skin. Accordingly, the expression of genes in the periderm and subperiderm differs significantly from gene expression in the postnatal epidermis, but gene expression in the inner layers of developing epidermis resembles that of mature epidermis. For example, the prototypical skin barrier gene *Filaggrin* is expressed during epidermal development, and the corresponding protein is processed in a similar manner as in the epidermis of the adult organism [79]. Likewise, loricrin is expressed in the granular layer of embryonic epidermis [59,80], and the upregulation of SPRR and LCE genes, reminiscent of stress responses of adult skin, can be increased prenatally by the deletion of the *Loricrin* gene [80,81,82]. The expression of several EDC genes of chickens and turtles was detected in developing skin [31,33], but only a few of them have been localized by mRNA in situ hybridization or immunohistochemistry within embryonic tissues of sauropsids. Comprehensive maps of EDC gene expression during embryonic and postnatal epidermal stratification are established by tissue transcriptomics, single-cell transcriptomics and spatial transcriptomic studies of skin development in phylogenetically diverse amniotes [83,84,85,86].

### 6.2. EDC Genes Expressed in the Embryonic Periderm or Subperiderm

Several genes of the EDC of different species of amniotes have been reported to be expressed in the embryonic periderm or subperiderm (Table 1). Here, we first discuss the evidence for the peridermal or subperidermal expression of SEDC-type genes and later discuss the expression of SFTP genes in the transient layers of the embryonic epidermis.

#### 6.2.1. Loricrin

Loricrin is encoded by an SEDC gene. The official gene symbol is now *LORICRIN*, whereas *LOR* was used previously in GenBank and in most publications until recently. In both mouse and human, loricrin is the main component of the cornified cell envelope [95,96]. Loricrin is highly enriched in glycine residues [20,97]. Its glutamine and lysine residues are target sites for cross-linking to SPRRs, other loricrin proteins and keratins filaments [20,98]. An ortholog of loricrin has been localized in the epidermis of the green anole lizard [31,99]. By immunolabeling with a carboxy-terminal specific antibody, it was detected in both the maturing “soft” alpha layer (lacunar cells) of scale epidermis and corneocytes of wound epidermis in the regenerating tail but not in the “hard” beta layer [99]. The lizard has two loricrin genes, and even three loricrin genes were identified in the chicken with each of them being expressed in both adult and embryonic skin [31]. By RT-PCR, loricrin mRNA was also detected in the skin of a turtle during embryonic development [33]. Loricrin was localized by fluorescent immunolabeling in the periderm of developing human skin [59]. At the ultrastructural level, immunogold electron microscopy detected loricrin in the cornified envelope of periderm cells [59].

#### 6.2.2. SPRRs

Small proline rich proteins (SPRRs) have a size of less than 20 kDa. They contain numerous proline-rich repeats and several glutamine and lysine residues which are sites of cross-linking to other proteins through the activity of transglutaminases [11,100]. The transcription of SPRR genes is upregulated in epidermal keratinocytes during wound healing [101]. Apart from their structural function in the cornified envelope, SPRRs have anti-oxidative properties [102]. SPRR2 and SPRR3 proteins were detected by immunofluorescence labeling during skin embryogenesis in humans. SPRR staining was observed in the periderm of the two-layered embryonic epidermis and in the embryonic spinous, granular and cornified epidermal layers at a later stage [59]. Many different classes of proline-rich proteins have been identified in all sauropsids, but no definitive orthology has been established [31,33,34,35,36]. EDC genes encoding SPRR-like proteins were reported to be expressed during the embyronic development in chickens and turtles [31,33].

#### 6.2.3. EDMTFH

*EDMTFH* is an SEDC gene located between the loricrin and CBP genes of the chicken genome. Its name stands for “Epidermal Differentiation Protein starting with an MTF motif and rich in Histidine” and indicates some of the few protein features that could be deduced from the amino acid sequence at the time of identification [31]. EDMTFH of the chicken has 99 amino acid residues of which 40% have aromatic side groups (F, W, Y, H). There are five EDMTF proteins in chicken, but only EDMTFH has a high content of histidine residues (>15%). A partial sequence of the protein was originally published as histidine-rich protein, HRP-B [103], leading to confusion with the mammalian SFTP, filaggrin, which is also rich in histidine residues [88]. *EDMTFH* belongs to a subcluster of gene EDC genes, initially named epidermal differentiation proteins starting with the MTF motif (EDMTFs) [31] and later renamed EDAAs (epidermal differentiation proteins rich in aromatic amino acids) [33,104]. EDMTF/EDAA genes underwent expansion by gene duplications in alligators [35], and genes of this family have been translocated to a non-EDC locus in turtles [33], whereas EDMTF/EDAA genes are not found in lepidosaurs (lizards, snakes and tuatara).

The EDMTFH protein was localized through both immunohistochemistry and immunogold labeling in the subperiderm of scutate scales and feather barb and barbules (E16–E19) of chickens [88]. It is expressed neither in the periderm nor in the normal epidermis. Electron microscopy indicated that EDMTFH partially co-localized with feather CBP bundles in barbule cells during the embryonic feather formation [88]. The results of the EDMTFH immunolocalization study supported the previously proposed evolutionary–developmental link between subperiderm and feather barb and barbules [51].

#### 6.2.4. EDCRP

Epidermal differentiation cysteine-rich protein (EDCRP) has an unusually high content of cysteine residues (36%) and is therefore considered a candidate for disulfide cross-linking [73]. It contains sequence repeats with the consensus sequence CCDPCQ(K/Q)(S/P)V. The sequence of avian EDCRP is similar to the sequences of cysteine-rich keratin-associated proteins (KRTAPs) of mammals, although these proteins are not phylogentically related [73]. Chicken EDCRP contains 385 amino acid residues among which paired cysteine residues (CC) are periodically found [73].

EDCRP was detected by RT-PCR in chicken embryonic skin, feathers and scales as well as adult feathers [31]. The expression in feathers was confirmed by a mass spectrometry-based proteomic analysis of chicken feathers [31]. In a later study, the expression of EDCRP was determined by mRNA in situ hybridization during embryonic development. EDCRP mRNA was detected in feather barbs and barbules of day E18 of embryonic development [73] and, importantly, in the subperiderm over the embryonic scutate scales [73]. The subperidermal keratinocytes were the only cells outside of feather follicles that were positive for EDCRP. Because of the above-mentioned sequence similarity with mammalian KRTAPs, it is conceivable that EDCRP is covalently linked by disulfide bonds to cysteine-rich keratins or other proteins to increase the mechanical resistance of cells [73]. The specific role of EDCRP in the subperiderm and the properties of the subperiderm require further investigations.

#### 6.2.5. EDDM

Epidermal Differentiation protein containing DPCC Motifs (EDDM) has even more cysteine residues than EDCRP and all other chicken EDC proteins [89]. The expression pattern of EDDM during skin development in chicken was studied using RT-qPCR and immunohistochemical analysis [89]. Immunolabeling with an EDDM antibody detected the protein in feathers [89] where EDDM was also detected by proteomics [31]. Furthermore, EDDM was detected by immunostaining in the subperiderm of embryonic scutate scales [89].

EDDM is conserved in birds, and orthologs of EDDM exist in crocodilians [35,89]. Of note, an ortholog of the other major avian cysteine-rich EDC protein, EDCRP, is also present in crocodilians [38]. The characteristic internal sequence repeats are conserved in the EDDM ortholog [89] but not in the EDCRP ortholog of alligators and crocodiles. As crocodilians develop a subperiderm, it will be very interesting to determine whether the subperidermal expression of EDCRP and EDDM is conserved in crocodilians.

#### 6.2.6. Corneous Beta Proteins (CBPs), Previously Referred to as Beta-Keratins

CBPs, formerly called beta-keratins, are characteristic and quantitatively dominant proteins of the epidermis in sauropsids. The name CBP indicates the expression in corneous skin structures and the ability to form a beta sheet (beta-pleated sheet). The latter trait depends on a conserved core domain of 34 amino acid residues [105,106]. Through face-to-face interactions between beta sheets, CBPs form dimers, and dimers subsequently form fibers. The terminal domains of CBPs are predicted to interact with other proteins [105,106]. The original name, beta-keratins, was meant to indicate the presence in the skin but is now considered misleading. CBPs are not related to keratins, which are members of the intermediate filament protein family [31,107].

The genes that encode CBPs have evolved as a subtype of SEDC genes [31] which are typically arranged in a clustered manner within the EDC of birds [92,108,109], crocodilians [93], turtles [33,110], lizards [32], geckos [36,111] and the tuatara [36]. The number of CBP genes ranges from 22 in crocodiles to more than 140 in chickens [106]. Based on the major expression sites and sequence similarities, CBPs have been classified into claw, scale, feather, feather-like and keratinocyte subfamilies [28,29,92,112]. In birds and turtles, the CBP genes underwent an expansion of copy numbers and gene translocations to loci outside of the EDC [33,108,109,110]. Feather CBP genes can be found on at least seven different loci of the chicken genome [108].

CBPs are expressed during chicken skin development, as demonstrated by both in situ hybridization [113,114,115] and immunolabeling [91,116,117]. Using an antibody raised against a scale CBP, CBPs were detected in the embryonic epidermal layers over developing scutate and reticulate scales as well as apteric skin of embryonic day 17, whereas only scutate scales were immunopositive for these CBPs in mature skin [91], which indicated a temporal and region-specific control of CBP expression during development. An antibody against feather CBPs revealed specific expression in the subperiderm over scutate scales, suggesting a homologous protein composition of feathers and subperiderm [58,117]. Subsequently, feather CBP homologs were also detected by immunostaining in the subperiderm of alligators. Together, these findings led to the hypothesis that the subperiderm originated in a common archosaurian ancestor and feathers coopted subperidermal proteins to the development of feathers [51,94].

Transcriptomic analyses showed that CBPs are differentially expressed in different types of embryonic chicken tissues, such as scales and feathers [110]. The expression sites were determined by mRNA in situ hybridization of chicken embryos [90]. CBPs named Chr25-FK12 and Chr27-FK12 and possibly Chr25-Claw9 were expressed in the subperiderm. However, the interpretation of CBP expression data is currently complicated because the nomenclature of CBPs and the nomenclature of embryonic skin layers is not consistently used by all research groups.

The embryonic skin development of crocodilians was investigated in several detailed histological and cytological studies. An antibody against chicken scale CBP (β1) was used to study epidermal development in alligators [49,51,94]. CBP expression was detected in the subperiderm of early stages of development (stages 24 and 25). The immunopositivity was maintained only in the definitive beta layer of stage 28 epidermis of hard outer (dorsal) scales but not in the ventral and hinge regions of scales [49,94]. By applying CBP gene-specific RT-PCRs, the expression of nine individual CBPs was detected in claws, the caruncle and scales at developmental stages 21, 23, 25 and 27, revealing a variation in CBP expression levels between stages and tissue types [93].

In lepidosaurs, CBPs were reported to be expressed during the development of the adhesive setae of geckos [118] and during skin development of the green anole lizard [32]. However, the expression of CBPs and other epidermal differentiation-associated proteins in the embryonic skin of all sauropsids except birds is largely unexplored. Further studies are necessary to integrate gene expression during development with hypotheses on the evolution of genes and skin in reptiles [33,51,58,90,92,93,108,110].

#### 6.2.7. Scaffoldin and Trichohyalin

Members of the SFTP family are expressed both during the initial formation of the stratum corneum and during the embryo-specific differentiation of the periderm. Filaggrin, filaggrin 2, hornerin and repetin are expressed in the developing epidermis of mouse embryos [79,119,120]. Trichohyalin and cornulin, which are evolutionarily more ancient than the aforementioned SFTPs, are not only expressed in immature epidermis and epidermal appendages but also in the periderm.

Trichohyalin, encoded by the gene *TCHH*, is mainly known as a protein of filiform papillae, the inner root sheath of the hair follicle and the medulla of hair shafts, where it forms intracellular granules, which are known as trichohyalin granules [13,121,122]. Trichohyalin interacts with keratin filaments via a short carboxy-terminal sequence motif that is conserved in many other SFTPs [76]. Scaffoldin (SCFN) is a homolog of mammalian trichohyalin in sauropsids [77]. It was named differently from trichohyalin because molecular phylogenetics based on S100 domain sequences of SFTPs did not unambiguously support orthology between trichohyalin and scaffoldin. However, the similarity between the two proteins is most striking in the segment following the S100 domain. Like trichohyalin, scaffoldin is extremely rich in the amino acid residues arginine (17% in alligator, 18% in chicken) and glutamic acid (17% in alligator, 19% in chicken) [77]. Also, the extreme length of scaffoldin of around 3200 amino acid (aa) residues in the chicken and 2678 aa in the alligator is similar to the length of human trichohyalin (1943 aa) [77].

SCFN is prominently expressed in the periderm of embryonic chicken skin [77]. It was specifically localized by mRNA in situ hybridization in the periderm but also in the subunguis of the claw [77]. At the protein level, SCFN was detected in the periderm of the skin leg (E18), where it localized to the periderm granules. The highest abundance of scaffoldin was observed in the multilayered periderm surrounding the egg tooth on the beak (E10) [60,77]. Further evidence for the expression of SCFN protein was obtained by immunoblotting, which revealed a band at the predicted size of 385 kDa in the embryonic beak, toe, skin, and tongue and an additional band reminiscent of the trichohyalin band that is linked to the deimination of arginine residues [77,123]. Immunogold electron microscopy indicated that scaffoldin forms the filamentous structures of periderm granules [50]. Periderm granules are not formed in the mammalian periderm, although some papers discussed the appearance of similar structures. However, trichohyalin was detected by immunohistochemical staining in the human periderm [87]. Recently, the expression of trichohyalin in the periderm was confirmed by single-cell transcriptomics of embryonic mouse skin [83]. These data suggest an evolutionarily conserved function of scaffoldin and trichohyalin in the periderm.

#### 6.2.8. Cornulin

Cornulin is an evolutionarily ancient SFTP that is expressed in the granular layer of the epidermis, the inner root sheath of hair follicles and the epithelium of the esophagus [124]. Like scaffoldin, cornulin is expressed in the periderm of embryonic chicken skin [58,77]. Immunolabeling of cornulin in embryonic mouse skin did not show expression in the periderm, but in the granular layer [77]. Of note, orthologs of cornulin exist in sauropsids and mammals, but cornulin is not conserved in green anole lizards, songbirds and whales [77,125,126]. These reported expression patterns suggest a partial redundancy of scaffoldin and cornulin in some but not all species of amniotes.

## 7. Conclusions

The accumulating evidence for the expression of EDC genes in embryo-specific layers of the epidermis, that is, the periderm and subperiderm, indicates functions of the EDC in controlling the development of the epidermis prior to its functions in the skin barrier and in the cornification of skin appendages of amniotes. This raises important questions regarding the molecular interactions of EDC proteins during development and the potential involvement in developmental defects due to aberrant or incomplete differentiation of the periderm [40]. Furthermore, the evolution of the EDC was linked not only to the adaptation to life in a dry environment [31] but also to new steps in the skin development of amniotes.

To better understand the functions of EDC proteins in skin development, detailed studies of gene expression, gene ablation or overexpression studies in phylogenetically diverse animal models and tests of protein interactions under defined conditions in vitro will be required. Together with detailed histological investigations, the identification of molecular markers of cell differentiation stages will help to close several important gaps in knowledge.

In conclusion, the recent progress in comparative genomics and gene expression studies have identified new roles of the EDC in skin development. Future studies will investigate the contribution of these roles of EDC genes in the phenotypic diversification of the skin during the evolution of mammals, reptiles and birds.

## Figures and Tables

**Table 1:** EDC genes expressed in embryo-specific epithelial skin layers.

Gene	Expression Sites	Protein Features *	References
Cornulin	Periderm (chicken)	Ca^2+^ binding, glutamine rich	[77]
Scaffoldin	Periderm (chicken)	Ca^2+^ binding, glutamic acid/arginine-rich	[50,77]
Trichohyalin	Periderm (human, mouse)	Ca^2+^ binding, glutamic acid/arginine-rich	[83,87]
Loricrin	Periderm (human)	Glycine-rich substrate of transglutamination	[59]
SPRRs	Periderm (human)	Proline-rich substrate of transglutamination	[59]
EDCRP	Subperiderm (chicken)	Cysteine-rich	[73]
EDMTFH	Subperiderm (chicken)	Histidine-rich	[88]
EDDM	Subperiderm (chicken)	Cysteine-rich	[89]
CBPs of feather-type	Subperiderm (chicken)	Beta-sheet, filaments	[25,51,58,90,91,92,93,94]

* For proteins with low sequence complexity, the predominant amino acid residues are indicated.
